# Examining Consequences Related to Unmet Care Needs Across the Long-Term Care Continuum

**DOI:** 10.1093/geroni/igaa057.296

**Published:** 2020-12-16

**Authors:** Meghan Jenkins Morales, Stephanie Robert

**Affiliations:** 1 University of Wisconsin, Madison, Wisconsin, United States; 2 UW-Madison, Madison, Wisconsin, United States

## Abstract

At some point in our lives, approximately 70% of us will need support to help with daily care. Without adequate assistance we may experience unmet care need consequences (UCNC) – such as skipping meals, going without clean clothes, or taking the wrong medication. This study examines the likelihood of experiencing UCNC related to gaps in assistance with activities of daily living (ADL) and instrumental activities of daily living (IADL) across long-term care arrangements: informal community care, paid community care, residential care, and nursing homes. We examine a sample of older adults receiving assistance in a care arrangement (N=2,499) from the nationally representative 2015 National Health and Aging Trends Study. Cross-sectional and longitudinal regression models, adjusting for differences in demographic and health/functioning characteristics, examine if type of care arrangement in 2015 is associated with UCNC in 2015 and change in UCNC by 2017. Holding all else constant, there were no significant differences in UCNC related to ADLs in 2015 across care arrangements. However, those receiving paid community care were more likely to experience UCNC related to IADLs (going without clean clothes, groceries, or a hot meal and making medication errors) compared to those receiving only informal care (OR=1.64, p<.05) or residential care (OR=2.19, p<.01). By 2017, paid care was also significantly associated with continued UCNC, but older adults in informal care arrangements were most likely to experience a new UCNC. Results suggest improving/expanding assistance with IADLs among community-dwelling older adults, and promoting equitable access to residential care, to reduce UCNC.

